# The Selection of Medical Books

**Published:** 1908-09-05

**Authors:** 


### The Medical Library.

### THE SELECTION OF MEDICAL BOOKS.

When the student of medicine who has passed his preliminary and intermediate examinations comes to consider the selection of a library such as his available funds allow, he soon discovers that certain principles must guide his choice with which he has not previously had to trouble himself. Hitherto his books have been the means to one end only, in the majority of cases; and that end is the acquisition of sufficient knowledge of physics, anatomy, and so on, to pass certain examinations. Broadly speaking his text-books are all discarded when he enters the wards, never probably to be opened again; though we must make an exception in regard to anatomical works, to which he should again and again have recourse before sitting for examination in surgery. But it is quite otherwise with text-books on medicine, surgery, and the main subdivisions of those subjects. It is only one function of the student's library now to assist him towards a qualification. Another, and if he realised it, more important is to furnish him with a sound practical knowledge of the profession he will have to practise for the rest of his working days.

No book can, of course, replace steady and painstaking work in the wards for this purpose; clinical experience must always be got in person to be of any real value, not vicariously supplied by another through the medium of paper and print. But wide reading must take a place if clinical facilities are to be turned to the best use. To investigate symptoms and signs is of but little use if it is not guided by the intelligent application of the knowledge gained by reading. The main difference between the case-taking of an experienced man and that of a clinical ward clerk is that the former soon picks the right line or lines to follow up, whereas the latter through lack of knowledge must laboriously pursue all in turn.

Now of the established favourites among text-books there are some which are frankly cram-books and little else, and there are some which, while valuable to one preparing for examination, are also sound and practical sources of information which will be of service in actual practice. Probably the best plan for the student at the beginning of his ward work is first of all to make himself acquainted with the details of clinical examination, physical signs and their import, clinical pathology, and so on; much of this he will be taught by his house physician, or should be, and he may supplement this by the study of one of the shorter books on clinical methods and examination. Meanwhile he will find it of advantage to read up the diseases of which instances are under his immediate notice in some standard book.

In the same way during his work in the surgical wards he may perfect himself in the use of dressings, the application of bandages and splints, and the technique of antiseptic and aseptic treatment, rather than the differential diagnosis of abdominal tumours let us say. Not that he need neglect any opportunities for laying the foundations of clinical experience by practising observation, adaptability and resource.

When he finishes his time as clerk, it is not a bad plan to get a comprehensive view of the outlines and inter-relations of disease generally, by reading through one of the more avowedly cram-books before he applies himself seriously to the digestion of a more worthy and dignified treatise. After that he may with advantage pass on to the study of the smaller monographs on special subjects, such as diseases of the heart and lungs, diseases of children, operative surgery, ophthalmic surgery, and so forth.

We should not advise him to purchase any book until he has read it, and in all cases not to buy examination books. It is in certain hospital libraries permissible to remove books at least for one night at a time, and thus to get a very good idea of the value of any book before deciding to purchase a copy of it.

The study of a good practical text-book which will be of service in after years is in reality, if it be done thoroughly, of more avail even for examination purposes than that of the elaborately tabulated and condensed examination guide book. The only advantage, and it is more apparent than real, more illusory than substantial, of the latter, is that it saves a certain amount of trouble for the time being; that is, it is possible for the student to say that he has "done" medicine or surgery, or what not in a fortnight or so, whereas he would obtain far more and far sounder knowledge by devoting a similar time to a fraction only of his subject in a better book. The student who neglects to look ahead and imagines that all his troubles will be over as soon as he is entitled to write a considerable fraction of the alphabet after his name, is common enough, but it is partly because his attention is not directed towards the larger aspects of his book-buying that his library is often unsuitable to his requirements after he has passed his examinations.

